# Breast cancer susceptibility gene 2 upregulation alleviated cardiac hypertrophy in angiotensin II-treated mice

**DOI:** 10.1016/j.bbrep.2026.102445

**Published:** 2026-01-19

**Authors:** Kun Liu, Xiao-Xuan Gong, Yong Li, Ming-Zhu Li, Chen Si, Lei Zhou

**Affiliations:** a Department of Cardiology, the First Affiliated Hospital of Nanjing Medical University, Nanjing, Jiangsu, China; b Department of Cardiology, The First People's Hospital of Lianyungang, Lianyungang, Jiangsu, China; c Department of Cardiology, Suzhou University Affiliated Taicang Hospital, Taicang First People's Hospital, Suzhou, Jiangsu, China

**Keywords:** Hypertrophic cardiomyopathy, Fibrosis, Inflammation, Apoptosis

## Abstract

Loss of breast cancer susceptibility gene 2 (BRCA2) function was found to exacerbate doxorubicin-mediated cardiomyocyte apoptosis and promote heart failure progression. We hypothesized that upregulation of BRCA2 may alleviate hypertrophic cardiomyopathy. Hypertrophic cardiomyopathy was established in mice via chronic angiotensin II (Ang II) administration (1.44 mg/kg/day) using osmotic minipumps. Cardiac BRCA2 expression was significantly downregulated in Ang II-treated mice. Cardiac hypertrophy triggered by Ang II in mice was significantly attenuated upon BRCA2 overexpression. Similarly, in cultured primary cardiomyocytes, Ang II-induced hypertrophic responses were suppressed by BRCA2 upregulation. The cardiac fibrosis was significantly attenuated after upregulation of BRCA2 in Ang II-induced hypertrophic cardiomyopathy. The myocardial inflammatory response to Ang II, characterized by elevated interleukin (IL)-1β, IL-6, and tumor necrosis factor alpha (TNF-α) levels, was markedly reduced with BRCA2 overexpression. The apoptotic biomarkers including Bax and cleaved caspase 3 (CC3) increased in the heart of hypertrophic cardiomyopathy, and attenuated after upregulation of BRCA2. These results indicated that upregulation of BRCA2 could improve hypertrophic cardiomyopathy. BRCA2 alleviated cardiac hypertrophy via attenuation of inflammation and apoptosis.

## Highlights

• BRCA2 expression was reduced in hypertrophic cardiomyopathy.
• Upregulation of BRCA2 could improve hypertrophic cardiomyopathy.
• BRCA2 attenuated hypertrophic cardiomyopathy through inhibition of inflammation and apoptosis.

## 1 Introduction

Pathological cardiac hypertrophy represents a major contributor to global disease burden and fatal outcomes. The development of cardiac hypertrophy, involving increased heart mass, stems from concurrent modifications in hemodynamic loading, biomechanical cues, and endocrine factors [1]. Cardiac remodeling in hypertrophic cardiomyopathy often involves progressive myocardial fibrosis as a key pathological component [2].

The breast cancer susceptibility genes (BRCA), BRCA1 and BRCA2, are the dynamic regulators of genomic integrity [3]. As a key DNA damage repair mediator, the tumor suppressor BRCA2 exhibits critical protective functions, with loss-of-function mutations conferring elevated risks for mammary and gynecological malignancies. In dilated cardiomyopathy, BRCA1 protein serves as an indicator of both widespread cardiomyocyte damage and compensatory upregulation through its antiapoptotic function [4]. The absence of functional BRCA2 protein potentiates doxorubicin-triggered programmed cell death in cardiomyocytes and worsens heart failure outcomes [5]. However, whether BRCA2 plays a role in hypertrophic cardiomyopathy is still precisely unclear.

Several proinflammatory cytokines, including interleukin (IL)-6, IL-18, transforming growth factor β (TGF-β) and nucleotide-binding oligomerization domain-like receptor proteins (NLRPs), contribute significantly to the inflammatory mechanisms underlying cardiomyopathy [6,7]. Previous study has shown that inflammatory cytokines increased in the TAC-induced cardiac hypertrophy [8]. Inactivation of BRCA2 not only induces tumor necrosis factor alpha (TNF-α) production but also heightens sensitivity to its pro-inflammatory actions [9]. This investigation evaluated the anti-inflammatory potential of BRCA2 overexpression in hypertrophic cardiomyopathy pathogenesis.

The apoptosis was enhanced in the heart of hypertrophic cardiomyopathy [10,11]. Cardiomyocyte hypertrophy induced by Ang II can be significantly attenuated after inhibition of apoptosis [12]. BRCA family members function in gene expression regulation, particularly targeting genes associated with cell proliferation control, DNA repair mechanisms, and apoptosis induction [13]. It is still unclear whether BRCA2 upregulation can attenuate cardiac apoptosis in mice with myocardial hypertrophy.

In summary, this work primarily addressed two interrelated questions: the functional impact of BRCA2 overexpression on hypertrophic cardiomyopathy progression, and its mechanistic involvement in regulating both inflammatory and apoptotic processes in cardiac hypertrophy.

## 2 Materials and methods

### 2.1 Animal ethics

The male C57/BL6/J mice were used in this study. The mice were obtained from Vital River Biological Co., Ltd (Beijing, China) and raised on a 12 h light-dark cycle with free access to standard chow and tap water. All experiments were approved by the Experimental Animal Care and Use Committee of Nanjing Medical University (Nanjing, China; 21050345) and followed the Guide for the Care and Use of Laboratory Animals (NIH publication No. 85–23, 1996).

### 2.2 Model of cardiac hypertrophy

To establish the cardiac hypertrophy model, 8- to 10-week-old male C57BL/6J mice were subjected to continuous angiotensin II (Ang II) delivery via subcutaneously implanted osmotic minipumps (ALZET, CA, USA). The pumps were surgically placed in the interscapular region and administered Ang II (Sigma-Aldrich, MO, USA) at a dosage of 1.44 mg/kg/day (dissolved in saline) or an equivalent volume of saline (vehicle control) for 4 weeks, with an infusion rate of 0.25 μl/h [14]. Simultaneously, BRCA2 overexpression was induced by injection of lentivirus (LV) via tail vein (LV-BRCA2; Genechem, Shanghai, China). The control mice were injected with LV-green fluorescent protein (LV-GFP).

### 2.3 Echocardiography determination

Four weeks post-infusion (Ang II or saline), left ventricular echocardiography was performed on anesthetized mice (1.5–2.5 % isoflurane) using a high-resolution imaging system (VisualSonics, Toronto, Canada). Key parameters, including left ventricular weight (LW), end-systolic interventricular septal thickness (IVSs), end-diastolic interventricular septal thickness (IVSd), left ventricular anterior wall thickness in diastole (LVAWd), left ventricular anterior wall thickness in systole (LVAWs), left ventricular posterior wall thickness in diastole (LVPWd), and left ventricular posterior wall thickness in systole (LVPWs), were recorded, with the mean values derived from three consecutive cardiac cycles.

Following echocardiographic assessment, mice were euthanized via cervical dislocation under deep anesthesia (2.5 % isoflurane). Heart weight (HW), HW-to-body weight (HW/BW) and HW-to-tibia length (HW/TL) ratios, as well as LW/BW, were determined. Cardiac tissues were either fixed in paraformaldehyde for histological analysis or snap-frozen and stored at −80 °C for further molecular studies.

### 2.4 Wheat germ agglutinin staining

Left ventricular samples were fixed in 4 % paraformaldehyde (24 h, room temperature), then sectioned and stained with FITC-conjugated wheat germ agglutinin (WGA; Invitrogen, CA, USA) to assess cardiomyocyte cross-sectional area. Fluorescence imaging was performed using a Zeiss microscope (Carl Zeiss GmbH, Germany), with quantitative analysis conducted using manufacturer-supplied software.

### 2.5 Masson staining

To evaluate cardiac fibrosis, 5-μm thick left ventricular sections were stained with Masson's trichrome (Servicebio, Wuhan, China) and visualized under a bright-field microscope (Carl Zeiss, Germany). Fibrotic areas were quantified using Image-Pro Plus (Media Cybernetics, MD, USA).

### 2.6 Immunofluorescence

Fixed left ventricular samples with 4 % paraformaldehyde at room temperature for 24 h. Then, incubated the samples with primary antibodies against BRCA2 (Abcam, MA, USA), IL-1β (Abcam), IL-6 (Abcam), TNF-α (Abcam), Bax (Abcam), or cleaved caspase 3 (CC3; CST, MA, USA) for a whole night at 4 °C. After that, incubated the samples with the relevant secondary antibodies (Abcam) for 2 h at room temperature. Afterwards, used 4’,6-diamidino-2-phenylindole (DAPI; Life Technologies Co., Grand Island, NY, USA) to counterstained nucleus. The images were obtained with a fluorescence microscope (Zeiss).

### 2.7 Cell culture and treatment

Primary neonatal rat cardiomyocytes (NRCMs) and cardiac fibroblasts (NRCFs) were isolated from 1-3-day-old Sprague-Dawley rats [15]. Briefly, atrial tissue and major vessels were removed, and the remaining myocardium was minced and enzymatically dissociated using PBS containing type II collagenase (Worthington Biochemical Corp., NJ, USA) and pancreatin (Sigma). Cell populations were separated by differential adhesion and cultured in DMEM supplemented with 10 % FBS and 1 % penicillin/streptomycin at 37 °C under 5 % CO_2_.

For phenotypic induction, NRCMs or NRCFs were transduced with LV-BRCA2 prior to 24-h stimulation with Ang II (10^−6^ M) to promote hypertrophic or fibrotic responses, respectively.

### 2.8 Quantitative real-time PCR analysis

Total RNA was extracted using TRIzol reagent (Invitrogen, USA) and reverse transcribed to cDNA with PrimeScript™ RT Master Mix (Takara Biotechnology) in a 10 μl reaction volume. qRT-PCR was performed in triplicate using the ABI Prism 7900 system (Applied Biosystems) with gene-specific primers (Table 1). Relative mRNA expression levels were calculated using the 2−ΔΔCt method, with normalization to GAPDH as an endogenous control.

**Table 1:** List of utilized primers for qRT-PCR.

Gene	Species	Forward primer	Reverse primer
Collagen I	Rat	TCAAGATGGTGGCCGTTAC	CTGCGGATGTTCTCAATCTG
Collagen III	Rat	CGAGATTAAAGCAAGAGGAA	GAGGCTTCTTTACATACCAC
TGF-β	Rat	CAGGGAGTAAGGGACACGA	ACAGCAGTTAGGAACCCAGAT
ANP	Rat	GAGCAAATCCCGTATACAGTGC	ATCTTCTACCGGCATCTCCTCC
BNP	Rat	GCTGCTGGAGCTGATAAGAGAA	GTTCTTTTGTAGGGCCTTGGTC
β-MHC	Rat	ACAATCCACGATGCAGAAGCT	GGGCCTTGGTCCTTTGAGA
GAPDH	Rat	GGCACAGTCAAGGCTGAGAATG	ATGGTGGTGAAGACGCCAGTA
Collagen I	Mouse	AAGAAGACATCCCTGAAGTCA	TTGTGGCAGATACAGATCAAG
Collagen III	Mouse	TTGGGATGCAGCCACCTTG	CGCAAAGGACAGATCCTGAG
TGF-β	Mouse	CGCAACAACGCCATCTATGA	ACTGCTTCCCGAATGTCTGA
ANP	Mouse	CCTAAGCCCTTGTGGTGTGT	CAGAGTGGGAGAGGCAAGAC
BNP	Mouse	AGACCCAGGCAGAGTCAGAA	CAGCTCTTGAAGGACCAAGG
β-MHC	Mouse	CTTCAACCACCACATGTTCG	TCTCGATGAGGTCAATGCAG
GAPDH	Mouse	CAAATTCCATGGCACCGTCA	GGAGTGGGTGTCGCTGTTG

### 2.9 Statistical analyses

Data are presented as mean ± SEM. All statistical analyses were performed using GraphPad Prism 7.0 (GraphPad Software, USA). For comparisons between two groups, unpaired Student's t-tests were used. Multiple group comparisons were analyzed by one-way ANOVA followed by Bonferroni post-hoc tests. Statistical significance was defined as P < 0.05 (∗P < 0.05, ∗∗P < 0.01, ∗∗∗P < 0.001, ∗∗∗∗P < 0.0001).

## 3 Results

### 3.1 The level of BRCA2

**Fig. 1:**
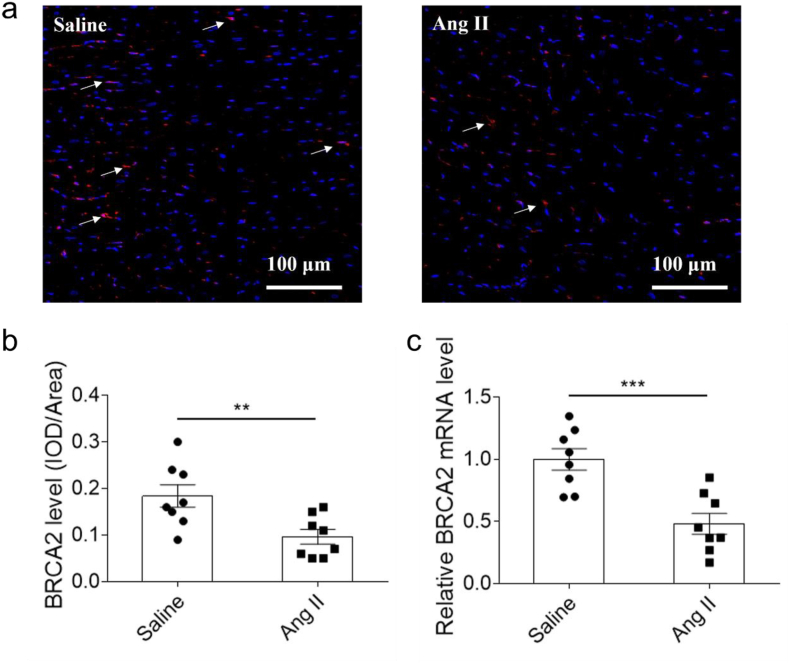
a, The protein level of BRCA2 reduced in the heart of mice treated with Ang II. b, The mRNA level of BRCA2 reduced in the heart of mice treated with Ang II. The results were expressed as mean ± SEM. n = 8 for each group. Unpaired Student's t-test was used. Bars, 100 μm. BRCA2, breast cancer susceptibility gene 2. Arrows, BRCA2.

Immunofluorescence analysis revealed a significant reduction in BRCA2 protein expression in cardiac tissue following Ang II treatment (Fig. 1a and b). Consistent with these findings, quantitative PCR demonstrated a corresponding decrease in BRCA2 mRNA levels in Ang II-infused mice compared to controls (Fig. 1c).

### 3.2 BRCA2 overexpression alleviated cardiac hypertrophy

**Fig. 2:**
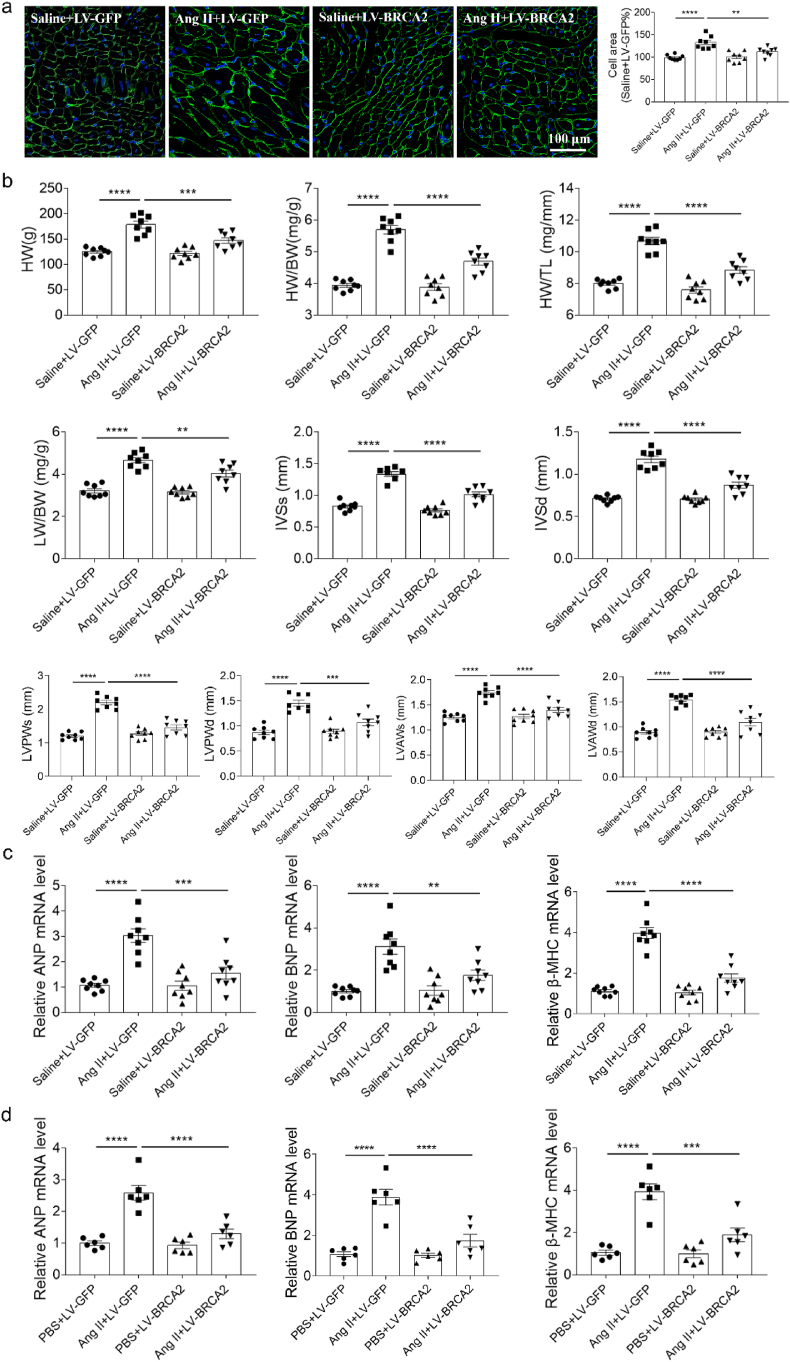
BRCA2 overexpression alleviated cardiac hypertrophy. a, BRCA2 overexpression inhibited the expansion of cardiocytes in the heart of mice induced by Ang II. b, BRCA2 overexpression inhibited the increases of HW, HW/BW, HW/TL, LW/BW, IVSs, IVSd LVPWs, LVPWd, LVAWs and LVAWd induced by Ang II of mice. c, BRCA2 overexpression inhibited the increases of ANP, BNP and β-MHC induced by Ang II in the heart of mice. d, BRCA2 overexpression inhibited the increases of ANP, BNP and β-MHC induced by Ang II in the cultured NRCMs. The results were expressed as mean ± SEM. n = 8 (a–c) or 6 (d) for each group. One-way ANOVA was used. Bars, 100 μm. BRCA2, breast cancer susceptibility gene 2; Ang II, angiotensin II; HW, heart weight; BW, body weight; TL, tibia length; IVSs, end-systolic interventricular septal thickness; IVSd, end-diastolic interventricular septal thickness; LVPWs, left ventricular posterior wall thickness in systole; LVPWd, left ventricular posterior wall thickness in diastole; LVAWs, left ventricular anterior wall thickness in systole; LVAWd, left ventricular anterior wall thickness in diastole; ANP, atrial natriuretic peptide; BNP, b-type natriuretic peptide; β-MHC, β-myosin heavy chain; NRCMs, neonatal rat cardiomyocytes.

WGA staining demonstrated that BRCA2 overexpression attenuated Ang II-induced cardiomyocyte enlargement in vivo (Fig. 2a). Quantitative analyses revealed that BRCA2 upregulation significantly suppressed Ang II-mediated increases in cardiac hypertrophy markers, including HW, HW/BW, HW/TL, LW/BW, ventricular wall thickness (IVSs and IVSd), left ventricular posterior wall thickness (LVPWs and LVPWd), and left ventricular anterior wall thickness (LVAWs and LVAWd) (Fig. 2b).

At the molecular level, Ang II-induced upregulation of fetal genes (atrial natriuretic peptide (ANP), b-type natriuretic peptide (BNP), and β-myosin heavy chain (β-MHC)) in cardiac tissue was markedly reduced by BRCA2 overexpression (Fig. 2c). This protective effect was consistently observed in vitro, where BRCA2 overexpression in NRCMs similarly blunted Ang II-stimulated expression of these hypertrophy markers (Fig. 2d).

### 3.3 BRCA2 overexpression alleviated cardiac fibrosis

**Fig. 3:**
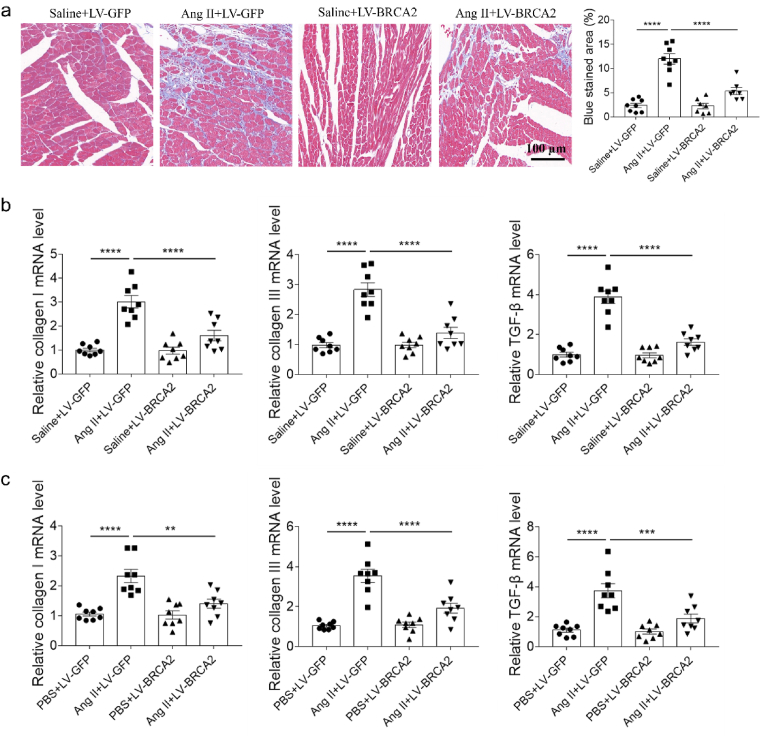
BRCA2 overexpression alleviated cardiac fibrosis. a, BRCA2 overexpression inhibited the cardiac fibrosis induced by Ang II of mice. b, BRCA2 overexpression inhibited the increases of collagen I, collagen III and TGF-β induced by Ang II in the heart of mice. c, BRCA2 overexpression inhibited the increases of collagen I, collagen III and TGF-β induced by Ang II in the cultured NRCFs. The results were expressed as mean ± SEM. n = 8 for each group. One-way ANOVA was used. Bars, 100 μm. BRCA2, breast cancer susceptibility gene 2; Ang II, angiotensin II; TGF-β, transforming growth factor β; NRCFs, neonatal rat cardiac fibroblasts.

Masson's trichrome staining revealed that BRCA2 overexpression significantly attenuated Ang II-induced cardiac fibrosis in mice (Fig. 3a). Molecular analysis demonstrated that Ang II-mediated upregulation of fibrotic markers (collagen I, collagen III, and TGF-β) in cardiac tissue was markedly suppressed by BRCA2 overexpression (Fig. 3b). This anti-fibrotic effect was recapitulated in vitro, with BRCA2 overexpression similarly preventing Ang II-induced elevation of these profibrotic factors in NRCFs (Fig. 3c).

### 3.4 BRCA2 overexpression alleviated inflammation of hypertrophic cardiomyopathy

**Fig. 4:**
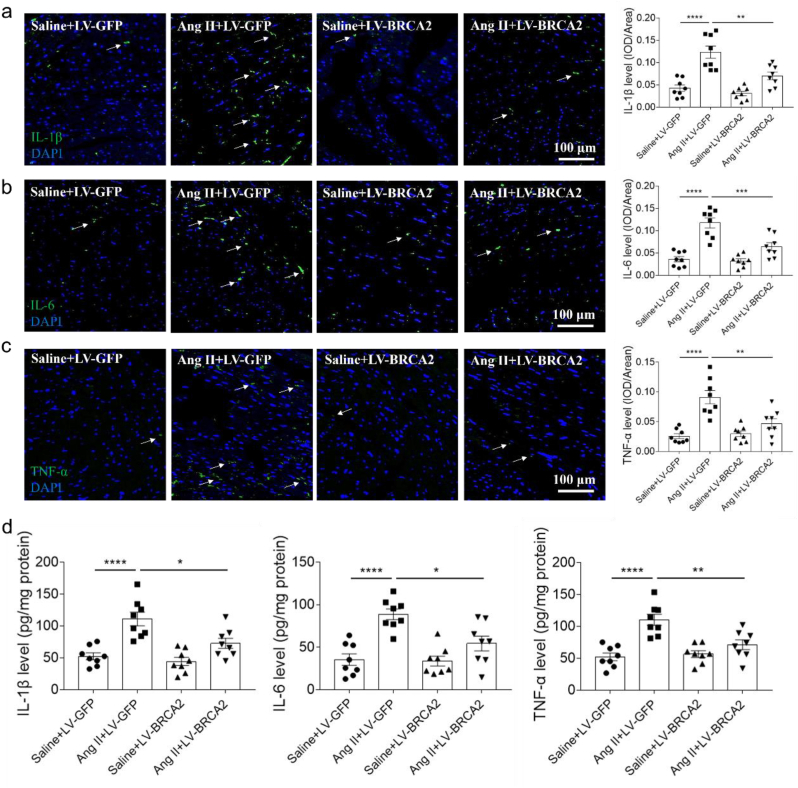
BRCA2 overexpression alleviated inflammation of hypertrophic cardiomyopathy. a, BRCA2 overexpression inhibited the increase of IL-1β induced by Ang II in the heart of mice through immunofluorescence detection. b, BRCA2 overexpression inhibited the increase of IL-6 induced by Ang II in the heart of mice through immunofluorescence detection. c, BRCA2 overexpression inhibited the increase of TNF-α induced by Ang II in the heart of mice through immunofluorescence detection. d, BRCA2 overexpression inhibited the increase of IL-1β, IL-6 and TNF-α induced by Ang II in the heart of mice through ELISA detection. The results were expressed as mean ± SEM. n = 8 for each group. One-way ANOVA was used. Bars, 100 μm. BRCA2, breast cancer susceptibility gene 2; Ang II, angiotensin II; IL-1β, interleukin-1beta; IL-6, interleukin-6; TNF-α, tumor necrosis factor-alpha; ELISA, enzyme-linked immunosorbent assay. Arrows, IL-1β (a), IL-6 (b) or TNF-α (c).

Immunofluorescence analysis revealed significant upregulation of pro-inflammatory cytokines (IL-1β, IL-6, and TNF-α) in Ang II-induced hypertrophic cardiomyopathy, which was substantially attenuated by BRCA2 overexpression (Fig. 4a–c). Complementary ELISA quantification confirmed that BRCA2 upregulation effectively blocked Ang II-mediated elevation of these inflammatory markers in cardiac tissue (Fig. 4d).

### 3.5 BRCA2 overexpression alleviated apoptosis of hypertrophic cardiomyopathy

**Fig. 5:**
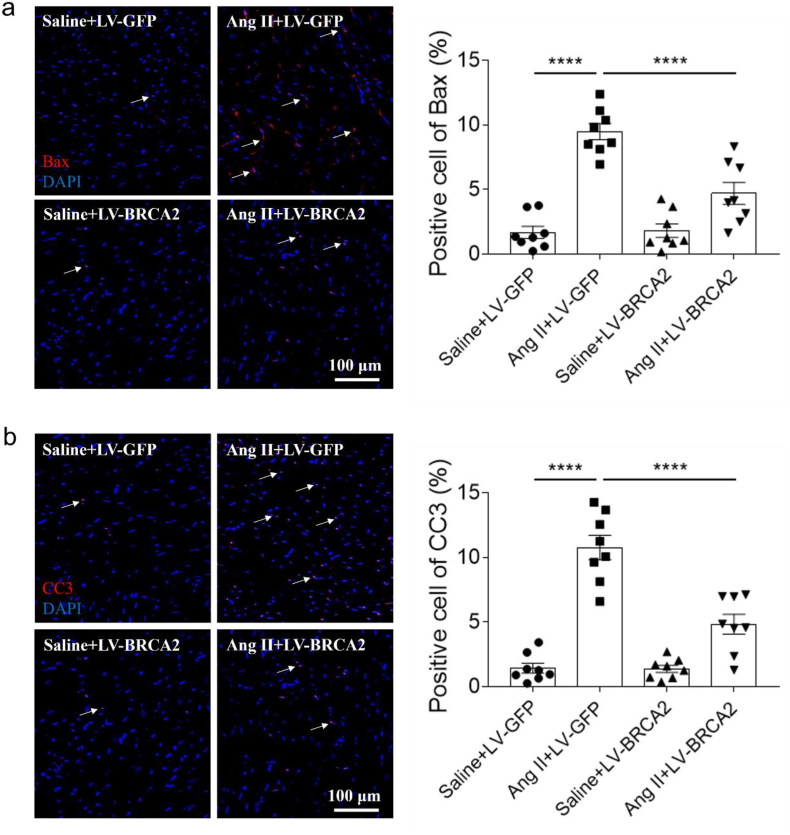
BRCA2 overexpression alleviated apoptosis of hypertrophic cardiomyopathy. a, BRCA2 overexpression inhibited the increase of Bax induced by Ang II in the heart of mice through immunofluorescence detection. b, BRCA2 overexpression inhibited the increase of CC3 induced by Ang II in the heart of mice through immunofluorescence detection. The results were expressed as mean ± SEM. n = 8 for each group. One-way ANOVA was used. Bars, 100 μm. BRCA2, breast cancer susceptibility gene 2; Ang II, angiotensin II; CC3, cleaved caspase 3. Arrows, Bax (a) or CC3 (b).

The level of Bax increased in the heart of mice with administration of Ang II, and this increase was inhibited by BRCA2 overexpression (Fig. 5a). In addition, the level of CC3 increased in the heart of mice with administration of Ang II, and this increase was suppressed by BRCA2 upregulation (Fig. 5b).

## 4 Discussion

The primary findings of this study were that BRCA2 expression is significantly downregulated in Ang II-induced cardiac hypertrophy. BRCA2 upregulation significantly attenuated both Ang II-induced hypertrophic cardiomyopathy and associated cardiac fibrosis. BRCA2 overexpression attenuated pathological cardiac hypertrophy through dual inhibition of inflammatory signaling and apoptosis.

It was reported that cardiac-specific BRCA1-deficient murine models revealed heightened cardiac vulnerability to both ischemic injury and doxorubicin-induced toxicity, suggesting broader implications for BRCA family proteins in cardioprotection against diverse stressors [16]. Our data demonstrate that chronic Ang II infusion significantly downregulates cardiac BRCA2 expression, correlating with hypertrophic progression, suggesting its potential role in pathological remodeling.

A report showed that BRCA1/2 mutation carriers exhibit an increased diabetes incidence alongside elevated cardiovascular risk profiles, suggesting shared metabolic pathophysiology [17]. Two distinct BRCA2 single nucleotide polymorphisms demonstrated significant associations with cardiovascular disease risk across diverse ethnic populations [18]. Our findings demonstrate that BRCA2 overexpression mitigates Ang II-induced cardiac hypertrophy and myocardial fibrosis, suggesting its protective role against hypertrophic cardiomyopathy and fibrotic remodeling.

Inflammatory pathways have been established as key drivers in the development and progression of hypertrophic cardiac remodeling [19], fibrosis [20], remodeling [21] and heart failure [22]. BRCA2 deficiency triggers TNF-α secretion and enhances cellular responsiveness to this pro-inflammatory cytokine, amplifying inflammatory signaling. [9]. However, the difference is that we found that overexpression of BRCA2 significantly suppressed the increases of inflammatory factors in the heart of Ang II-treated mice with Ang II treatment. The results illustrated upregulation of BRCA2 could attenuate the inflammatory responses of hypertrophic cardiomyopathy.

Progressive ventricular remodeling and cardiac functional decline are mechanistically linked to cardiomyocyte apoptotic pathways [23,24]. Cardiac functional decline and subsequent heart failure pathogenesis are driven by the gradual attrition of cardiomyocytes via apoptotic and necrotic pathways [25]. Programmed cardiomyocyte death emerges as a pivotal mechanism driving post-infarction cardiac remodeling processes [26]. The increase of apoptosis in left ventricular sections induced by doxorubicin was further significantly enhanced in cardiomyocyte-specific BRCA2 knock-out mice [5]. Our experimental data demonstrate that cardiac-specific BRCA2 overexpression effectively suppresses Ang II-induced upregulation of pro-apoptotic markers (Bax and cleaved caspase-3) in the mice models. These results demonstrated that BRCA2 alleviated hypertrophic cardiomyopathy through attenuation of apoptosis.

In conclusions, BRCA2 expression was reduced in hypertrophic cardiomyopathy. Upregulation of BRCA2 could significantly alleviated cardiac hypertrophy and the related fibrosis of heart. BRCA2 attenuated hypertrophic cardiomyopathy through inhibition of inflammation and apoptosis.

## Ethics approval statement

This study was approved by the Institutional Animal Care and Use Committee of Nanjing Medical University.

## Funding

Not applicable.

## CRediT authorship contribution statement

**Kun Liu:** Investigation, Project administration. **Xiao-Xuan Gong:** Data curation. **Yong Li:** Methodology. **Ming-Zhu Li:** Resources. **Chen Si:** Resources. **Lei Zhou:** Conceptualization, Writing – original draft, Writing – review & editing.

## Declaration of competing interest

The authors declare no competing interests.

## Figures and Tables

**Table:** 

Ang II	angiotensin II
ANP	atrial natriuretic peptide
BNP	brain natriuretic peptide
BRCA	breast cancer susceptibility gene
BW	body weight
CC3	cleaved caspase 3
GFP	green fluorescent protein
HW	heart weight
IL	interleukin
IVSd	end-diastolic interventricular septal thickness
IVSs	end-systolic interventricular septal thickness
LV	lentivirus
LVAWd	LV anterior wall thickness in diastole
LVAWs	LV anterior wall thickness in systole
LVPWd	LV posterior wall thickness in diastole
LVPWs	LV posterior wall thickness in systole
MHC	myosin heavy chain
NRCFs	neonatal rat cardiac fibroblasts
NRCMs	neonatal rat cardiomyocytes
TL	tibia length
TNF	tumor necrosis factor

## Data Availability

Data will be made available on request.
